# Predictions Based on Different Climate Change Scenarios: The Habitat of Typical Locust Species Is Shrinking in Kazakhstan and Xinjiang, China

**DOI:** 10.3390/insects13100942

**Published:** 2022-10-17

**Authors:** Rui Wu, Jing-Yun Guan, Jian-Guo Wu, Xi-Feng Ju, Qing-Hui An, Jiang-Hua Zheng

**Affiliations:** 1 Key Laboratory of Oasis Ecology of Xinjiang, Institute of Arid Ecology and Environment, College of Geography and Remote Sensing Science, Xinjiang University, Urumqi 830046, China; 2 College of Tourism, Xinjiang University of Finance and Economics, Urumqi 830012, China; 3 Locust and Rodent Control Headquarters of Xinjiang Uygur Autonomous Region, Urumqi 830001, China

**Keywords:** locust, climate change, maximum entropy model, China and Kazakhstan, risk management

## Abstract

**Simple Summary:**

We use maximum entropy (MaxEnt) model, modelling to predict habitat change and inform on potential prevention and control of locust disasters. Our research work is based on three kinds of migratory locusts in a niche overlap and the geographic distribution of preliminary forecast of the locust, the geographical distribution of typical quantitative habitat suitability change, understanding the China–Kazakhstan border areas, the concentrated distribution area of typical locusts and its future expansion area. The scope of the initial assessment of typical locusts confirms the emphases of the locust control between the two countries in the region, to provide a basis for future monitoring work. Large-scale locust monitoring measures are difficult to implement in practice; therefore, this study will provide a regional monitoring focus for locust control efforts. Our study complements this by assessing priority areas for prevention.

**Abstract:**

Climate change, especially climate extremes, can increase the uncertainty of locust outbreaks. The Italian locust (*Calliptamus italicus* (Linnaeus, 1758)), Asian migratory locust (*Locusta migratoria migratoria* Linnaeus, 1758), and Siberian locust (*Gomphocerus sibiricus* (Linnaeus, 1767)) are common pests widely distributed in the semidesert grasslands of Central Asia and its surrounding regions. Predicting the geographic distribution changes and future habitats of locusts in the context of climate warming is essential to effectively prevent large and sudden locust outbreaks. In this study, the optimized maximum entropy (MaxEnt) model, employing a combination of climatic, soil, and topographic factors, was used to predict the potential fitness areas of typical locusts in the 2030s and 2050s, assuming four shared socioeconomic pathways (SSP126, SSP245, SSP370, and SSP585) in the CMIP6 model. Modeling results showed that the mean area under the curve (AUC) and true statistical skill (TSS) of the MaxEnt model reached 0.933 and 0.7651, respectively, indicating that the model exhibited good prediction performance. Our results showed that soil surface sand content, slope, mean precipitation during the hottest season, and precipitation seasonality were the key environmental variables affecting locust distribution in the region. The three locust species were mainly distributed in the upstream region of the Irtysh River, the Alatao Mountain region, the northern slopes of the Tianshan Mountains, around Sayram Lake, the eastern part of the Alakol Lake region, the Tekes River region, the western part of Ulungur Lake, the Ili River, and the upstream region of the Tarim River. According to several climate projections, the area of potential habitat for the three most common locust species will decrease by 3.9 × 10^4^–4.6 × 10^4^ km^2^ by the 2030s and by 6.4 × 10^4^–10.6 × 10^4^ km^2^ by the 2050s. As the climate becomes more extreme, the suitable area will shrink, but the highly suitable area will expand; thus, the risk of infestation should be taken seriously. Our study present a timely investigation to add to extensive literature currently appearing regarding the myriad ways climate change may affect species. While this naturally details a limited range of taxa, methods and potential impacts may be more broadly applicable to other locust species.

## 1. Introduction

Locust outbreaks are often international, transboundary, and mobile. The damage caused by locusts results in economic losses for the agriculture and livestock industries, accelerates the rapid loss of vegetation cover, and disrupts the balance of grassland ecosystems [1,2]. The Italian locust ((*Calliptamus italicus* (Linnaeus, 1758)), Asian migratory locust (*Locusta migratoria migratoria* Linnaeus, 1758), and Siberian locust ((*Gomphocerus sibiricus* (Linnaeus, 1767)) are common pests in steppe, desert, and semidesert regions with long-distance migratory characteristics. The species share some similarities in bioecology and population dynamics, and therefore some overlap in habitat selection [3,4]. The Italian locust is often found from Ukraine to southern Siberia, eastern Kazakhstan, and northwestern China, while the Asian locust and Siberian locust have a much wider range, covering the entire temperate and steppe regions of Eurasia, causing severe damage to local steppe ecosystems and agricultural production [5,6,7]. The occurrence of locust infestations is continuous and results from a combination of environmental factors and ecological conditions. Outbreaks of some pests, such as locusts, have profoundly affected human life; for example, desert locusts swept through the Horn of Africa and southwest Asian countries in 2020 [8,9]. In addition, swarms of the Italian locust and the Moroccan locust (*Dociostaurus maroccanus* (Thunberg, 1815)) damaged crops in Russia and China, respectively [10]. In the Lake Balkhash region, Asian migratory locust outbreaks occur at random intervals of two to ten years and can persist for two to ten years [11]. The Ili Delta is a permanent breeding ground for Asian locusts, with over 30% percent of the region affected [12]. In certain wetlands in Xinjiang, China, the high summer temperatures and humidity provide the ideal circumstances for Asian migratory locusts to lay eggs and hatch.

The border between China and Kazakhstan is more than 1700 km long. Since 1980, more than 20 large-scale migratory events have occurred in the study area, with an average economic loss of approximately 14 million RMB per event [13]. Starting in 1980, there were more than 10 major locust migrations in six years; large migrations occurred in 1999, 2000, and 2008 [14,15,16]. In Kazakhstan, locusts migrated from the border to the Tacheng basin after consuming vegetation in the local pastures; this led to continuous large and high-density locust infestations, resulting in significant ecological and economic losses. The grassland ecosystem is one of the most important ecosystem types in northwestern China and northern Kazakhstan, and it has a significant impact on local ecological changes and human activities in the region [17,18]. A number of intergovernmental agreements have been signed between countries to address the transboundary migration of locusts, including a round table entitled “Locust Control Problems in Central Asia”, organized by the Kazakhstan Institute of Strategic Studies in February 2001 and a series of intergovernmental agreements signed between Kazakhstan and China in Beijing in December 2002 [16]. The Kazakhstan–China Locust Control Group carried out joint locust survey work in 2014, 2015, 2018, and 2019. Locusts are the most important animal species in grassland ecosystems, and their species diversity and the resulting changes in population growth and community structure, especially the occurrence of locust infestations due to population outbreaks, are crucial to the structure, function, health, and maintenance of grassland ecosystems. Extensive ploughing often results in a very significant decrease in locust abundance, especially in steppe regions. In the mid-1950s, vast steppe areas were ploughed, and many remaining steppe habitats became overgrazed [19]. As a result, many locust habitats have been destroyed or damaged. Locusts may enhance net primary production over the years. For example, in dry steppes and semideserts, even between outbreaks, the Italian locust can consume a significant part of the aboveground vegetation, damaging the growing points of sagebrushes and accelerating their growth [5,20]. In addition, the plants themselves are harmed as locusts consume seeds and sprouts, inhibiting natural regeneration. However, in the solitary stage, the presence of locusts is beneficial to the ecosystem; they facilitate nutrient cycling and play an important role in the food chain [21]. Controlling locust outbreaks is therefore crucial to land management and maintaining food security. Predicting future suitable locust habitats enables relevant agencies to implement scientifically based, accurate locust control measures and stay abreast of locust occurrences and developments Monitoring can be increased in areas with possible high-density outbreaks. Early warning information can be issued in a timely manner to guide effective prevention and control efforts and to prevent large-scale locust outbreaks from causing food losses and damage to the grassland environment.

Climatic factors have been shown to affect the embryonic development of species, outbreak occurrence, and biological interactions [22,23]. The IPCC Sixth Assessment Report (AR6) highlighted the localized effects of global warming. Global temperatures are projected to increase by 2 °C to 3 °C by 2100, with a median change of 2.2 °C. According to the IPCC, five shared economic pathways predict future climate change: the green growth paradigm (SSP1), intermediate development pathways along with historical patterns (SSP2), regionally heterogeneous development (SSP3), geographically and socially unequal development (SSP4), and development pathways dominated by high demand for energy provided by fossil fuels (SSP5). Kazakhstan and Xinjiang, China, have typical semiarid and arid climates; arid regions are more vulnerable to persistent climate change and weather extremes. Changes in climate and weather may directly affect crop pest conditions and increase the frequency of insect outbreaks.

Using remote sensing, modern locust hazard monitoring can map locust habitats and observe locust population dynamics on scales ranging from the neighborhood level to the country level. Ref. [12] mapped of migrating locust habitats in Asia, particularly in the Ili River Delta, has been conducted by using Landsat images, which may be extended to other regions where migratory locusts threaten agricultural productivity. Ref. [24] developed a multisensor monitoring system for assessing the risk of locust damage in the Lake Balkhash basin. Ref. [21] fused a multiscale approach to model habitat suitability index (HSI) maps for three different locust species. Since the 1980s, research on locust prediction has improved in a number of ways due to the development of remote sensing techniques [25,26,27,28]. The availability of new datasets (e.g., soil moisture), methods, and technological progress contribute to this steady improvement. Species distribution models are binary models based on machine-learning algorithms used to understand the current distribution of species [5,29,30]. Various modeling approaches and MaxEnt model predictions are widely used in species distribution studies because they can accurately simulate species distributions and maintain accuracy and robustness in small sample settings [30,31,32]. Several researchers have predicted locust habitats based on species distribution models [9,33,34]. Ref. [35] used the MaxEnt model to predict areas at high risk of infestation by the Siberian locust and found them to be mainly located on the northern border and the northern slope of Tianshan Mountain. Ref. [36] used bioclimatic data combined with the MaxEnt model to predict the habitat range of Italian locusts in Xinjiang from 2021 to 2040 (the 2030s), 2041 to 2060 (2050s), and 2061 to 2080 (2070s), assuming various future scenarios (RCP2.6, RCP4.5, and RCP8.5). The results showed that the distribution pattern of the Italian locust fitness zone in northern Xinjiang and the Tianshan Mountains remained unchanged in the BCC_CSM1.1 scenario. Ref. [13] used the CLIMEX model, set to predict the Asian flying locust habitat based on a future warming scenario, and the results showed that the species were distributed in the northern Xinjiang (42.69°–48.29° N) and Kazakhstan (47.03°–51.65° N) regions.

In previous studies, only climatic factors were selected as environmental variables. Soil factors that could affect locust reproduction and egg incubation were not considered; these soil factors require further investigation. Most previous studies were based on a single global climate model (GCM) and a single locust species. However, there are some similarities among locust species in terms of their response to climatic conditions, grassland types, and soil environments. There are similarities in soil conditions, vegetation types, and locust species occurring in the Tacheng locust-bearing area in China and the Alla Lake locust-bearing area in Kazakhstan. There have been no substantial differences in the average annual temperature and precipitation over the last 30 years. Therefore, we selected the Italian locust, Asian migratory locust, and Siberian locust as typical locust-representative species in Kazakhstan and Xinjiang, China, to evaluate the suitability of the area as a locust habitat and the potential outbreak risk.

The GCM structure is a source of uncertainty in climate change projections. Previous studies have shown that multimodel integration (MME) can sufficiently reduce the uncertainty of simulated climate conditions, compared to individual GCM models, and reduce the influence of climate patterns on the models, thus improving the prediction accuracy [33,37,38]. The principle of “one model, one vote” in MME has become the most popular strategy for reducing uncertainty in independent models. In MME, all GCMs are integrated with equal weight, and utilizing more GCM data leads to more accurate integration results [39]. Therefore, including multiple environmental variables in the GCM climate model can effectively reduce the error in the model fitting and make the model more reliable.

In the context of global warming and anthropogenic trends, combined with current state-of-the-art climate models, our study had the following aims: (1) to explore critical environmental variables affecting typical locusts; (2) to predict the potential geographic distribution of typical locusts in Kazakhstan and Xinjiang, China; (3) to quantify changes in habitat suitability of typical locusts in the 2030s and 2050s based on different climate change scenarios; and (4) to provide a preliminary assessment of potential typical locust outbreak risk.

## 2. Materials and Methods

### 2.1. Species Occurrence Data

Occurrence data for Italian locusts, Asian migratory locusts, and Siberian locusts were obtained from different sources: (1) 11 records were obtained from the Global Biodiversity Information Facility (GBIF, https://www.gbif.org/ (accessed on 1 January 2022.)); (2) a total of 1526 records were obtained by the research team from multiyear field sampling of GPS data in locust occurrence areas; and (3) 99 occurrence records were extracted from published literature [13,20,21,40]. In total, we obtained 1613 locust occurrence sites, including 931 for Italian locusts, 576 for Siberian locusts, and 106 for Asian flying locusts. We cleaned the occurrence data by removing records with duplicate close occurrences and removing missing georeferenced records.

**Figure 1:**
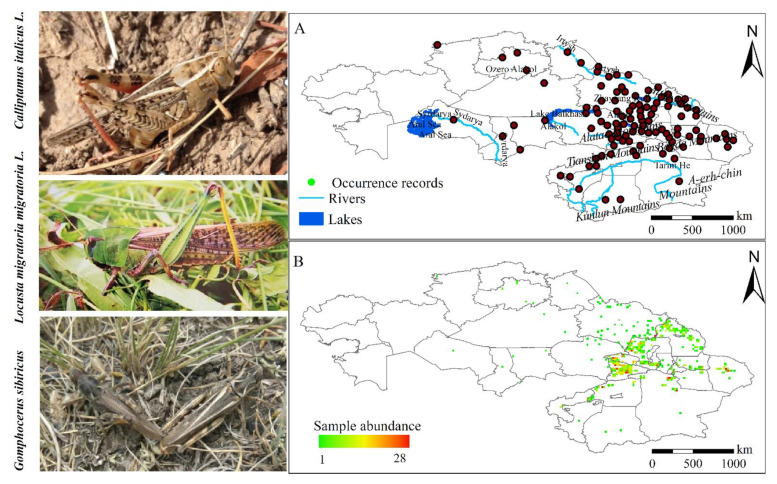
(**A**) Screened sample records. (**B**) Spatial richness of typical locust samples when unscreened.

To avoid model overfitting, we first reduced spatial autocorrelation by spatially filtering occurrence data within the study area using the SDMtoolbox tool “Spatially Sparse Occurrence Data” to eliminate spatial clustering of locations for model calibration and evaluation [41]. Spatial filtering was used to minimize omission errors and commission errors, and ultimately, 109 occurrence records for the three locust species were used for potential locust distribution predictions based on historical and future climate conditions (Figure 1A).

### 2.2. Environmental Variables

The environmental datasets used were bioclimatic and elevation data and soil data. Bioclimatic and elevation data were obtained from WorldClim version 2.1 climate data (https://worldclim.org/accessed on 10 January 2022) at a spatial resolution of 2.5 arc-minutes. Soil data were obtained from the World Soil Database at a spatial resolution of 30 arc-seconds (http://www.fao.org/ (accessed on 10 January 2022)). The historical climate data we used included 19 bioenvironmental variables measured between 1970 and 2000 and one topographic variable: elevation data. Slope and slope direction data were generated in ArcGIS 10.2 based on elevation data. The future climate variables were obtained from CMIP6 projections for the periods between 2021 and 2040 and between 2041 and 2060. We adopted a multimodel integration approach to reduce uncertainty in the climate models and used bioclimatic data from 20 GCMs for our projections: ACCESS-ESM1-5, BCC-CSM2-MR, CanESM5, CMCC-ESM2, CNRM-CM6-1, CNRM-CM6-1-HR, CNRM-ESM2-1, EC-Earth3-Veg, EC-Earth3-Veg-LR, GISS-E2-1-G, GISS-E2-1-H, INM-CM4-8, INM-CM5-0, and IPSL-CM6A-LR. The topography was based on future projections, and the soil data were consistent with historical periods. Bilinear interpolation was used to resample all environmental variables to 0.5° × 0.5°.

Environmental variables are important parameters in the construction of ecological niche models. Using too many environmental variables to build a model will enhance the spatial correlation between variables, resulting in overfitting, and thus reduce the transferability of the model. On the contrary, choosing a moderate and reasonable number of environmental variables can significantly improve the prediction ability of the model [29,33]. Therefore, it is necessary to calculate the correlation of environment variables and exclude those variables with high correlation. When the Pearson correlation coefficient is higher than 0.75, we define it as a highly correlated variable. The Pearson correlation and variance inflation factors (VIFs) were used to select environmental variables, and the first 23 environmental variables were rescreened (Table S1). First, jackknife analysis was selected in the MaxEnt model to test the contribution of the environmental variables. Variables with correlations greater than 0.75 were removed based on variable contribution value analysis. VIFs were used for assessments of the covariance between variables [41,42]; variables with VIF < 10 were retained, and ultimately, nine environmental variables were used for modeling, including seven bioclimatic variables (Table 1).

### 2.3. Modeling Process

The suitability of habitats for the three locust species was assessed based on current and future climate scenarios. species records, and environmental variables [31]. In the MaxEnt (v3.4.4k, http://biodiversityinformatics.amnh.org/open_source/MaxEnt/ (accessed on 15 January 2022.)) version, 25% of the samples were selected for testing, and 75% were selected for training the model. To avoid overfitting, 120 combinations of six model element types (L = linear, Q = quadratic, H = hinge, P = product, T = threshold) and a regularization multiplier (values ranging from 0.5 to 10 with an increment of 0.5) were used to optimize the performance of the model [33,43,44]. Selection of optimal modeling parameters was based on the area under the subject operating characteristic (ROC) curve and AUC values. Ten repetitions were performed using a repetition-type bootstrap approach. The maximum number of iterations was 1000, and the threshold type known as “maximum training sensitivity plus specificity” (MTSS) was applied for binary prediction.

Among the 23 environmental variables initially selected, nine variables were screened for use in the MaxEnt model modeling. There were four categories based on the thresholds; Areas with values less than the threshold were considered unsuitable for locust growth, and values above the threshold indicated low suitability, moderate suitability, and high suitability. Areas were classified into four categories by comparing the distribution of the locusts in the future based on current and alternative climate scenarios: unsuitable (0–MTSS), low suitability (MTSS–0.4), moderate suitability (0.4–0.6), and high suitability (>0.8). Based on environmental data from 20 models and assuming four different scenarios for the periods between 2021 and 2040 and between 2041 and 2060, the predictions were equally weighted and averaged to obtain the average predicted probability of potential future habitats.

### 2.4. Model Evaluation

We used the receiving operator characteristic (ROC) area under the curve (AUC) values and true skill statistic (TSS) to evaluate model performance. The AUC values ignore the predicted probability values and increase the average weight omission and commission errors. Therefore, we introduced the TSS statistic, which is a threshold-dependent accuracy measure that retains all the advantages of the kappa statistic while compensating for its disadvantages [45,46]. AUC values range from 0 to 1, and larger AUC values represent better model fit. TSS values between 0 and 0.6 indicate poor model performance, and values between 0.6 and 0.7, between 0.8 and 0.9, and between 0.9 and 1 indicate fair, good, and excellent model performance, respectively [47]. The TSS is an intuitive measure of performance for species distribution models; predictions are expressed as presence-nonexistence maps, with TSS values between −1 and +1. The closer the TSS value is to 1, the better the model accuracy [45,48]. The AUC and TSS values of the model increase when the species’ geographic range is extended beyond its current range.

To reduce the uncertainty of the prediction results, we introduced the majority voting method, which superimposes the results of model runs in different periods to obtain the final prediction results for locust habitats.

## 3. Results

### 3.1. Model Evaluation and Contribution of Variables

In this study, we used the AUC values of the ROC curve to assess the performance of the species distribution model. Additionally, TSS values were assessed to improve the performance of the model. When the default RM of the model was 1 and the feature combination was LQHP, the model had an AUC value of 0.933 and a TSS value of 0.7651. The model exhibited maximum performance when the feature combination was LQHPT and the RM was 0.5, the AUC of the model was 0.955, and the TSS value was 0.8115 (Table S2). The AUC and TSS values of the optimized model were greater. The average values of the AUC and TSS indicated that the predictions of the MaxEnt model were accurate.

**Figure 2:**
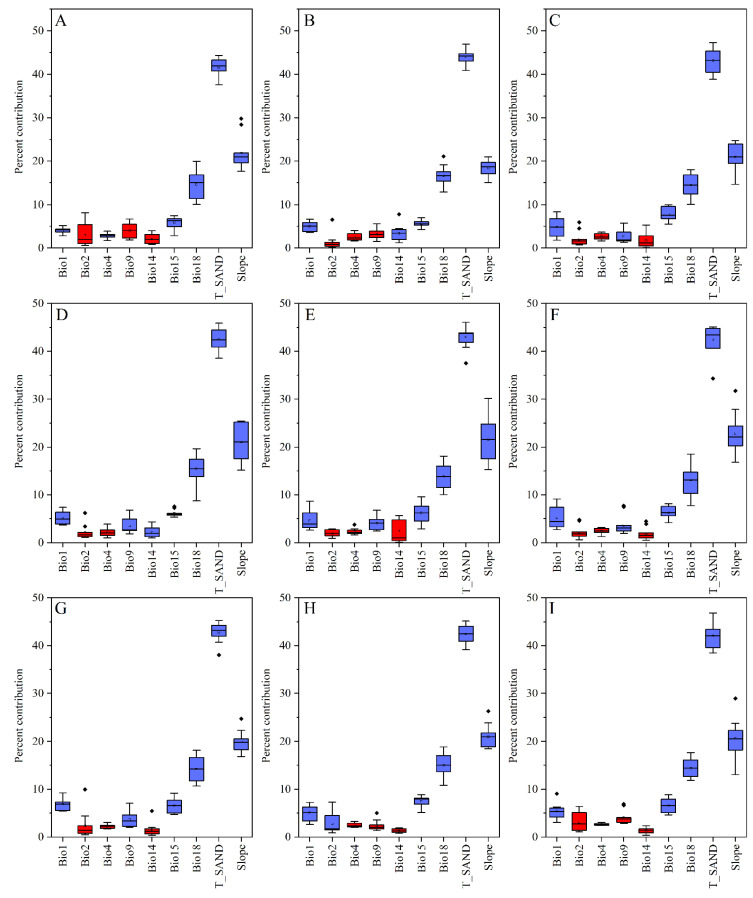
(**A**)**–**(**I**) Represent the percentage contribution of nine environmental variables (Bio1, Bio2, Bio4, Bio9, Bio14, Bio15, Bio18, T-SAND and Slope) in the MaxEnt model for the current time period and for different paths in the 2030s and 2050s (SPP126, SSP245, SSP370, SSP585). The box plots show the median (horizontal line inside the box), first and third quartiles (upper and lower edges of the closed box), and the maximum and minimum values (ends of the box whiskers). Outlier values more than 1.5 times the interquartile range are depicted as circles, and values more than three times the interquartile range are denoted as stars. The blue boxplot represents environmental variables with high contribution rates, and the red boxplot represents environmental variables with low contribution rates.

The predicted percent contribution of environmental variables after 10 replication runs based on different climate scenarios was assessed. The variables T-SAND, slope, bio18, bio15, bio9, and bio1 contributed relatively significantly to the model (Figure 2A–I).; therefore, these variables had the greatest impact on the predictions of the MaxEnt model. Of these variables, T-SAND, slope, bio18, and bio15 had a total contribution to the model of more than 80% (Figure 2A–I).

### 3.2. Habitat Suitability under Current Conditions

**Figure 3:**
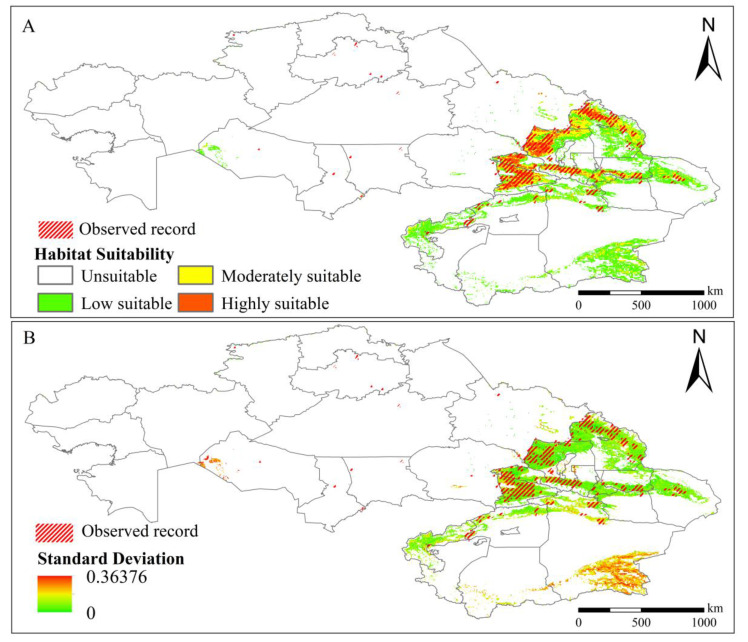
Prediction of potential habitats for typical locusts under current conditions. (**A**) Predicted probability of occurrence. (**B**) Standard deviation in 10 replicate tests.

Figure 3A shows that the predicted range of potentially suitable habitats coincided with the recorded occurrence records and was concentrated along the northern slopes of the Tianshan Mountains in Xinjiang, China. The areas of high suitability in Xinjiang, China, were mainly distributed in the upper reaches of the Irtysh River, the Alatao Mountain region, the northern slopes of the Tianshan Mountains, the Sairim Lake region, the eastern part of the Ala Lake region, and the Teix River region; the areas of moderate suitability were distributed in the periphery of the high-suitability region, specifically in the western part of Ulungu Lake, the northern slopes of the Tianshan Mountains, and Sairim Lake. Low-suitability areas were distributed around moderate-suitability areas, specifically in Alatao Mountain, the Khairat River, the area of Ulungu Lake, the northern slope of Tien Shan, the area of the upper Tarim River, and the area of the A-erh-chin Mountains. In addition, most of the areas in Kazakhstan were unsuitable for locust survival. Only a few areas of low suitability were distributed around Lake Zaysan, Lake Balkhash, the Irtysh River and areas near Lake Alakol and the Aral Sea.

The standard deviations across model runs were modest, indicating that the model is resilient. The largest standard deviations were for the A-erh-chin Mountains and Aral Sea regions, indicating that the estimated possible habitat ranges in those areas were inaccurate (Figure 3B). The model classified areas with uncertainty as having low or moderate suitability, although no locust occurrences were recorded in those areas (Figure 3A).

### 3.3. Habitat Suitability under Future Conditions

**Figure 4:**
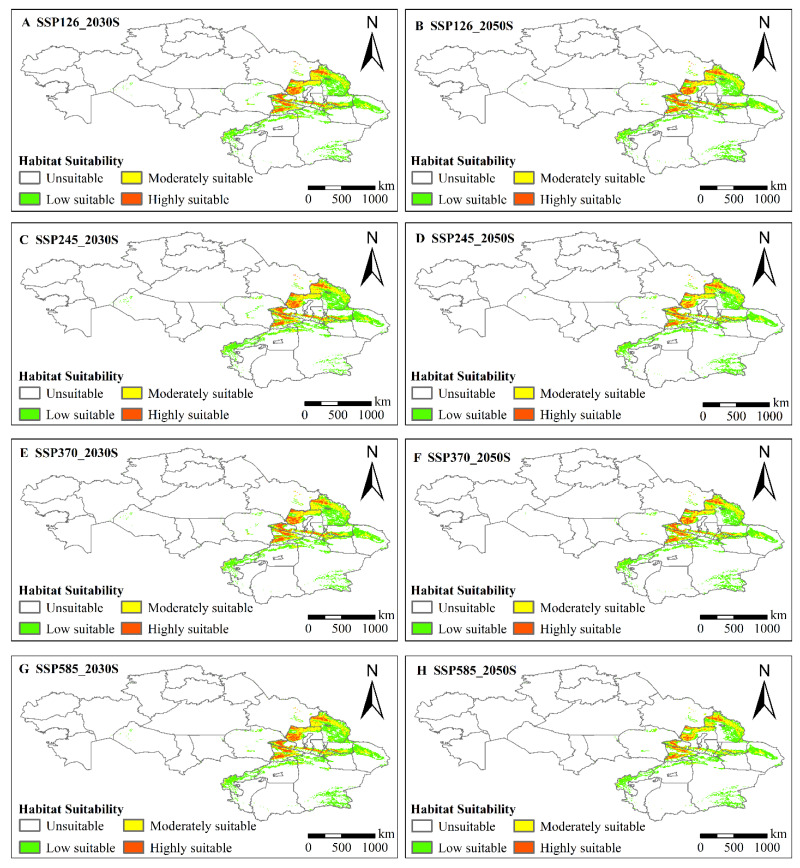
(**A**–**H**) Typical locust potential habitat projections for two time periods (the 2030s and 2050s) under four climate scenarios (SSP126, SSP245, SSP370, and SSP585).

The MaxEnt model predicted the potential distribution of locusts in the 2030s and 2050s. Compared to the current situation, the predicted potential habitat ranges, assuming different scenarios for the two periods, were generally consistent in spatial distribution (Figure 4A–H).

**Figure 5:**
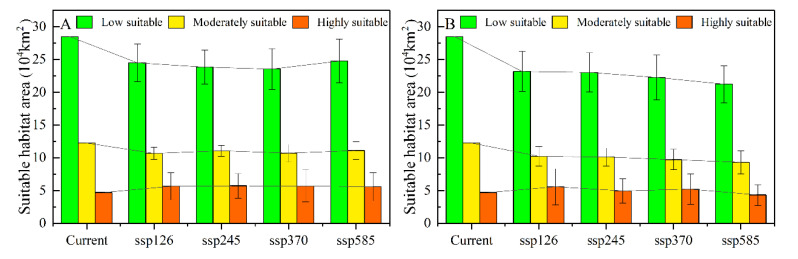
Area of typical locust suitable habitat assuming different scenarios. (**A**) 2030s, (**B**) 2050s. The error bars represent the standard deviation between the results of the 20 GCM models.

**Figure 6:**
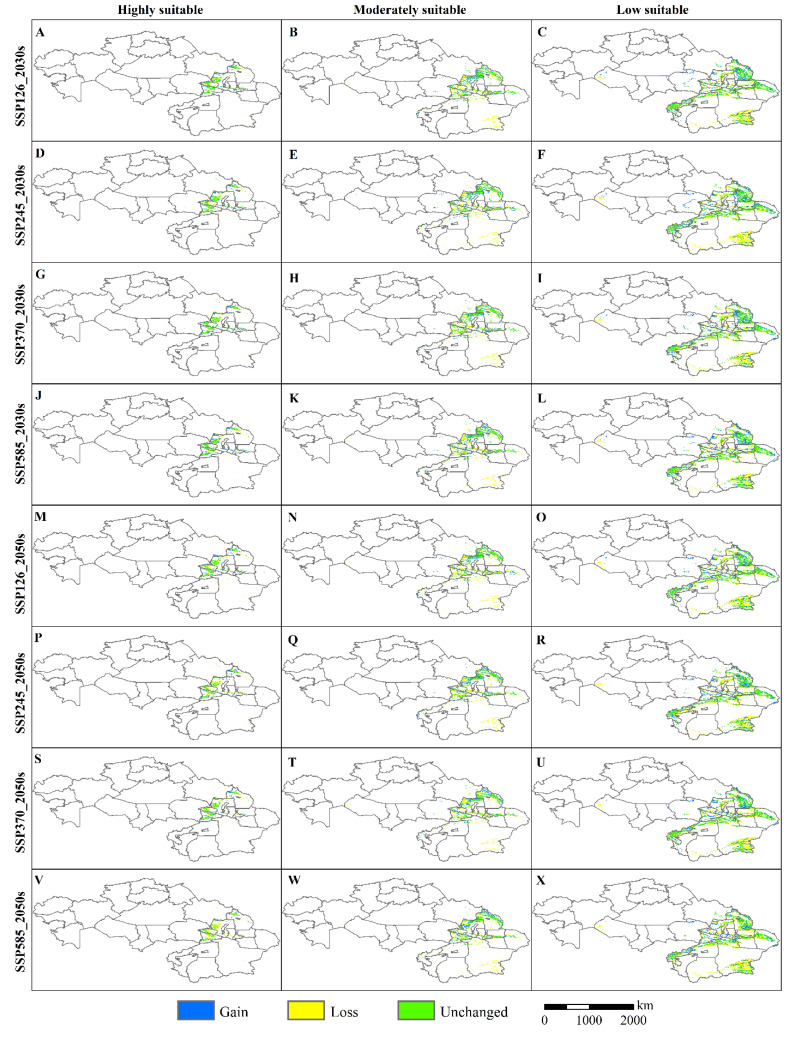
(**A**–**X**) Changes in potential habitats of typical locust species relative to current habitats based on different climate scenarios in the 2030s and 2050s.

Specifically, in all periods, the potentially suitable habitat areas showed an overall contraction in all scenarios. The moderate- and low-suitability regions decreased in area to different degrees. In contrast, the high-suitability regions increased in area to different degrees. The increase was mainly due to the transfer and spread of low- and moderate-suitability areas to high-suitability areas (Figure 4A–H, Figure 5A–B). In future climate projections, the expansion of high-suitability areas for locusts was mainly dispersed along the Irtysh River, in the western part of the Ulungu Lake area, the eastern part of Alakol Lake, and the northern slope of the Tianshan Mountains, and contracted in the upper reaches of the Irtysh River, the upper Tarim River area, the area around the Ili River, and A-erh-chin Mountains (Figure 6A–V). The expansion of the moderate-suitability area was in the southern part of the Altai Mountains, Ulungu Lake, the northern slope of the Tian Shan, the Kashgar River, Bogda Mountain, and the Lake Balkhash region, and the contraction was in the Bosten Lake Basin, the northern slope of the Tian Shan, the upper Tarim River, and around the A-erh-Chin Mountain range (Figure 6B–W). The expansion of the low-suitability area was scattered along the Ulungu River, Bogda Mountains, upper Tarim River, the A-erh-chin Mountains, the Ili River, and near Lake Balkhash, and contracted in the lower Ulungu River, the marginal area of the Tuha Basin, A-erh-chin Mountains region, Kunlun Mountains, and the region near the Aral Sea (Figure 6C–X).

Compared with the current situation, the areas of low and moderate suitability for the three typical locusts in Kazakhstan and Xinjiang, China, decreased. The areas of high suitability exhibited a transient increasing trend in the 2030s and then exhibited a decreasing trend in the 2050s. Based on different climate scenarios, the habitat area of the three typical locusts decreased by 3.9 × 10^4^–4.6 × 10^4^ km^2^ in the 2030s and by 6.4 × 10^4^–10.6 × 10^4^ km^2^ in the 2050s (Figure 5A–B). The low-suitability area decreased by 2 × 10^4^–3.3 × 10^4^ km^2^ in the 2030s and by 3.0 × 10^4^–4.7 × 10^4^ km^2^ in the 2050s; the moderate-suitability area was reduced by 1.7 × 10^4^–1.9 × 10^4^ km^2^ in the 2030s and by 1.7 × 10^4^–2.7 × 10^4^ km^2^ in the 2050s. The high-suitability area increased by 0.1 × 10^4^–0.4 × 10^4^ km^2^ in the 2030s and decreased to 0.4 × 10^4^–1.6 × 10^4^ km^2^ in the 2050s. By comparing the suitable habitats of the three typical locust species based on different climate scenarios, the areas of high suitability are expected to decrease by 1.3 × 10^4^–1.7 × 10^4^ km^2^ in the 2030s (Table 2). Based on different climate scenarios, the habitats of three typical locust species decreased by 3.9 × 10^4^–4.6 × 10^4^ km^2^ in the 2030s and by 6.4 × 10^4^–10.6 × 10^4^ km^2^ in the 2050s (Table 2).

## 4. Discussion

### 4.1. Influence of Environmental Factors on the Potential Distribution of Typical Locusts

**Figure 7:**
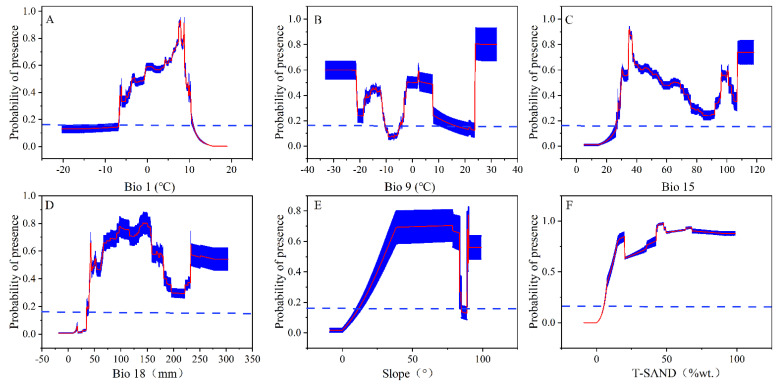
Response curves for the six most critical environmental variables. (**A**)–(**F**): Bio1, Bio9, Bio15, Bio18, Slope, T-SAND. The red curve and blue shading indicate the mean response and mean +/− one standard deviation for the 10 MaxEnt replicate runs. The blue dashed line indicates the maximum training sensitivity plus specificity threshold.

By comparing the contribution of environmental variables in the MaxEnt model based on current and future climate scenarios, we found that the factors that contributed the most were T-SAND, slope, bio18, bio15, bio9, and bio1) (Figure 2A–I). This indicated that these environmental variables significantly influenced the potential geographic distribution of locusts. The response curves of the variables reflected the range of adaptation of locusts to these environmental variables, and the range of adaptation of locusts to the six environmental variables at the MTSS threshold was as follows: T-SAND (12–100% wt.), slope (12–84°), bio18 (38–180 mm), bio15 (30–120), bio9 (−35–10 °C, −6.5–17 °C, 24–33 °C), and bio1 (−7.5–12.5 °C) (Figure 7A–F).

The locust habitat is not static, and understanding its dynamics requires a comprehensive consideration of the various environmental elements and the similarities among locust habitats. Our data indicated that soil sand was a crucial factor affecting locusts, and studies have shown that locusts prefer to lay eggs in moderately dense sandy soils rather than in loose or dense soils [11]. It has been reported that locusts tend to lay eggs in soils with salinities of 0.2% to 1.2% and moisture contents of 15% to 18% in loamy soils (10% to 12% in sandy soils and 18% to 20% in clay soils) [49]. Soils with a fine texture and high pH are also suitable for spawning Asian migratory locusts [40]. Asian migratory locusts are mostly found around rivers and lakes. When the water level in a lake decreases, the beach area increases, and the groundwater table rises, providing suitable conditions for locusts to lay eggs and hatch. For example, high summer temperatures and humidity create suitable conditions for Asian migratory locusts to lay eggs and hatch around some marshes in Xinjiang, China.

Temperature may affect the hatching, worm, and adult stages, potentially shifting the timing of developmental stages to earlier in the year and shifting and expanding the range of locust infestation. Egg overwintering is the species’ annual cycle’s most critical and vulnerable stage. In winter, the temperature drops, and the locust eggs are dormant. Oviposition occurs from late April to June, and the low-temperature threshold for egg development is thought to be 16 °C. Eggs usually develop faster at higher temperatures, and temperatures between 20 °C and 42 °C are suitable for worm development. At lower temperatures, it will take longer to complete each developmental stage [50,51]. Temperature significantly affects insect development, and the appropriate, sensitive, and stressful temperatures for the development of Siberian locusts are 21 to 24 °C, 27 to 30 °C, and greater than 32 °C, respectively. The Italian locust has a more robust tolerance to high temperatures than the Siberian locust. The appropriate, sensitive, and stress temperatures for the growth of Italian locusts are 27 to 30 °C, 33 to 39 °C, and greater than 41 °C, respectively [52]. Ref. [53] showed that the egg stagnation rate of locusts could reach 90.7% at 22 °C and 16 days at a short photoperiod of 8 L. Treatment at 13 °C for 20 days was effective in terminating egg stagnation. Locusts prefer warmer sites and start to move at 15 °C on sunny days, and more activity is observed at 35 °C. High temperatures and low rainfall in summer create favorable conditions for a high frequency of locust winging and long-sustained migration [15]. Our results showed that the average temperature of the driest season suitable for locusts is 24 to 33 °C. Hot season precipitation (bio18) and average annual temperature (bio1) limit locust development. This result was consistent with that of previous studies predicting the potential distribution of locusts in Siberia [35]. That study showed that the average winter temperature was high and precipitation was low in years with severe locust infestation. The higher winter temperature facilitated the safe overwintering of locust eggs. When the temperature increased and precipitation decreased, the population size increased. The Yili region, Tacheng, and Tianshan areas, which are most suitable for locusts, have typical arid climate characteristics, with mean annual temperatures of −4 to 8 °C and an annual precipitation of 100 to 200 mm [54].

The causes of locust infestation are not only related to temperature and humidity; topography is also one of the main factors influencing the occurrence and regional distribution of locusts. As altitude increases, light, temperature, humidity, and soil structure also change significantly, resulting in different vegetation characteristics at different altitudes, leading to a clear vertical distribution pattern of vegetation. The vertical distribution of vegetation at different altitudes is distinct. The changes in vegetation, temperature, and humidity lead to a variety of food sources and habitats for locusts, resulting in the presence of different locust communities at different altitudes. The development of locusts varies according to altitude and climatic conditions. Reports indicated that locusts take 20 to 25 days from hatching to emergence in the mountain steppe areas of the western Tien Shan at 2500 to 2700 m, and 25 to 31 days in the alpine steppe areas at 2500 to 3000 m. In nature, the emergence of each generation of locusts generally takes 5 to 7 days in subalpine grasslands and 5 to 10 days in alpine grasslands. Previous studies have shown that locusts inhabiting desert and semidesert grasslands, such as the Italian locust, are more tolerant of high temperatures, while locusts inhabiting alpine and subalpine grasslands, such as the Siberian locust, are less tolerant of high temperatures. The differences in tolerance to high temperatures are the result of long-term adaptive evolution, and with the continued warming conditions in Central Asia, the spatial distribution patterns of locust taxa in different habitats will change significantly. Locust taxa inhabiting desert semidesert grasslands and alpine subalpine grasslands will gradually move to higher altitudes and higher latitudes as the temperatures increase; this phenomenon is consistent with our findings [13,36]. In summary, our results were consistent with previous conclusions based on experience.

### 4.2. Changes in the Geographic Distribution of Typical Locusts under Climate Change

We used the MaxEnt model to simulate the relationship between specific locust breeding sites and key bioclimatic and soil sand content variables in Kazakhstan and Xinjiang, China. Changes in climatic conditions significantly impact pest development, reproduction, survival, and natural enemies. More frequently than plants and vertebrates, insects respond to climate change because of their physiological characteristics, short reproduction time, and high reproduction rate. The tendency of locust habitat contraction became more pronounced with increasing radiation intensity. Notably, the contraction of the 2050s was more evident in the SSP585 scenario. Previous studies have shown that in different scenarios, Central Asia is expected to experience more drought events of longer durations, but a lower intensity in the future in the period from 2021 to 2050 [55,56]. However, they will occur more frequently for longer durations and with greater severity and intensity in the distant future. In the low-emission scenario (SSP126), drought events occur more frequently than under the high-emission scenario (SSP585) [55]. Central Asia may experience a “dry side dry, wet to wet” future. Previous studies suggested that in the middle of the century, temperatures in Xinjiang will generally increase by between 1.5 °C and 2 °C, with relatively large warming in the Junggar Basin hinterland and the Hami Basin. At the end of the century, the temperature increase in Xinjiang will be between 4 °C and 6 °C, with a spatial distribution similar to that in the middle of the century [57]. The percent increase in precipitation and the value of increase in precipitation in Xinjiang in the middle of this century are expected to be between 8% and 10% and from 25 mm to 39 mm, respectively, and the percent increase in precipitation and the value of increase in precipitation at the end of this century are projected to be between 19% and 39% and from 75 mm to 137 mm, respectively; the percent change of the large valuable area is still dominated by the basin [58]. The abovementioned areas of environmental degradation correspond to areas where the potential habitat of typical locusts has been reduced (the upper reaches of the Irtysh River, the Bosten Lake Basin, the northern slopes of the Tianshan Mountains, the marginal area of the Tuha Basin, Margins of the Tarim Basin, and the Upper Tarim River). Locust habitat changes will be influenced by future climate change, and when the magnitude of future climate change exceeds the locusts’ survival threshold, the area suitable for locust survival will shrink. Sustained high temperatures and precipitation may destroy locust breeding and foraging habitats, so areas of moderate and low suitability for locust survival will decrease in the middle of the 21st century.

Our study showed that the suitable areas for the three typical locust species are expected to expand in the upper reaches of the Irtysh River, the Alatao Mountains, the northern slopes of the Tianshan Mountains, Sayram Lake, the eastern part of Alakol Lake, the Tekes River, the western part of Ulungu Lake, the Ili River, the upper Tarim River region, and the area around the A-erh-chin Mountains. The precipitation will be relatively high in mountainous areas such as the Tianshan Mountains, Kunlun Mountains, and Altai Mountains, with significant wetting changes [55]. Suitable conditions of temperature, humidity, and precipitation are conducive to the hatching and development of locusts, so the areas mentioned above are likely to be suitable locust habitats. Most of the regional localities in Kazakhstan are primarily unsuitable for breeding and establishing locusts. There are no areas of high suitability for the three typical locust species in the territory. However, [13], using the CLIMEX model, it was predicted that high-suitability areas for Asian migratory locusts are located in the northern part of East Kazakhstan and the southeastern part of the Almaty Oblast [13], which is slightly different from our conclusion.

Our study suggests that locust occurrence is significantly associated with contemporaneous climate warming in Central Asia. Kazakhstan and Xinjiang, China, are among the midlatitude dryland regions. Northern Kazakhstan has experienced a 35% decrease in precipitation and a 1 to 2 °C average temperature increase in recent decades, and the rate of warming has been significantly higher than the Northern Hemisphere average [59]. In response to warming conditions, locust taxa in different habitats in Kazakhstan and Xinjiang, China, will exhibit extensive changes in their spatial patterns. The expansion of the highly suitable zone was mainly scattered in the Irtysh River, the Ulungu Lake region, and along the eastern part of western Alla Lake and the northern slopes of the Tianshan Mountains.

Although droughts will become less frequent in the future, they will be longer in duration and more intense and are expected to become more severe in northwestern China and western Uzbekistan [60]. With global warming conditions, this will lead to more extreme weather. For example, in Central Asia, extremely high temperatures may reach 40 °C in the low elevation plains and shallow temperatures below −50 °C in the high mountains [58]. Semiarid and arid regions have fragile ecosystems and are more vulnerable to persistent climate change and extremes. Under two °C (RCP4.5-2040-2059), drought conditions will worsen in Kazakhstan and Xinjiang in northwestern China [61]. Climate change, especially in its extremes, can affect species distributions. Studies have shown that climatic extremes cause many locust infestations. The large locust infestation in the Sino-Kazakhstan border region between 1999 and 2001 was also associated with a warm winter and an extensive drought [62]. Although the potential locust habitat is shrinking, future climate extremes in the region are increasing; thus, the suddenness of locust infestations should be considered.

### 4.3. Uncertainty and Outlook

**Figure 8:**
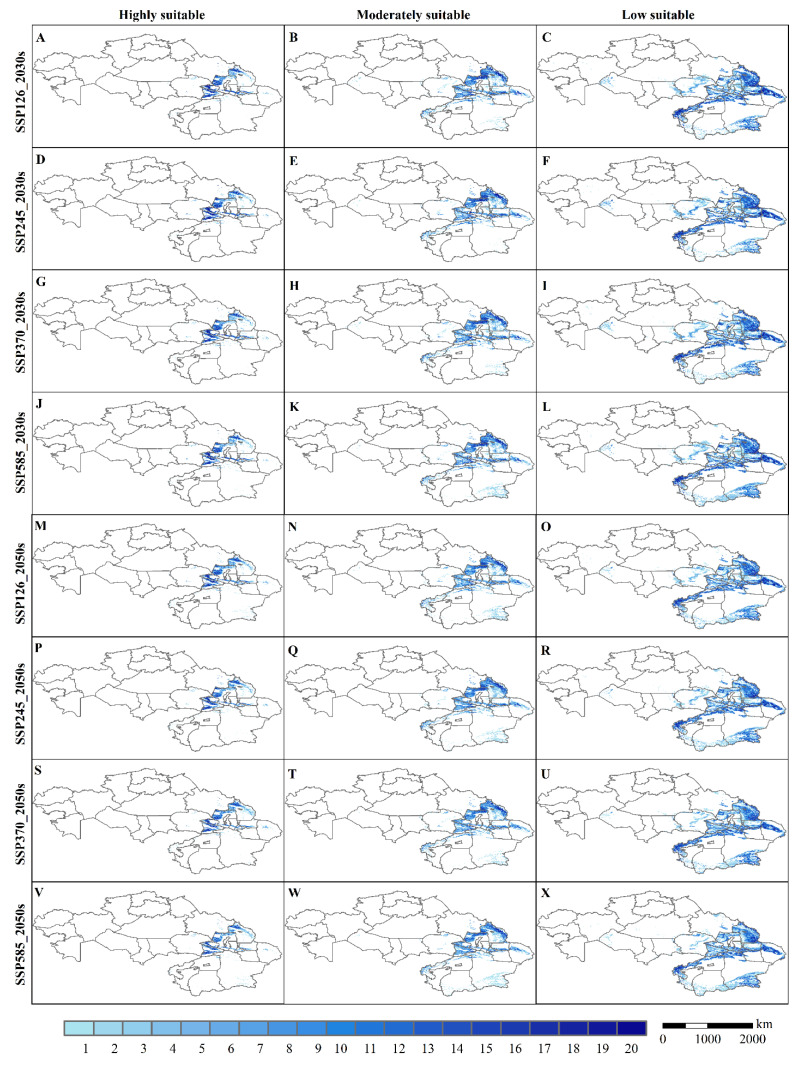
(**A**–**X**) Consensus map of suitable areas for typical locusts in the 2030s and 2050s based on climate data for 20 GCMs. The values (1–20) indicate the number of voting consensuses. Larger values indicate higher consistency of simulation results.

Figure 8A–X shows the consensus map of potentially suitable areas for the three typical locust species predicted in the 2030s and 2050s. Overall, the uncertainty is lower for highly suitable areas and moderately suitable and higher for less suitable areas. The above indicates the high accuracy of our forecast results. The uncertainty is mainly in the Aljinshan, upper Tarim River in Xinjiang, China. We used the MAXENT model to predict the fitness zones of three typical locust species in Xinjiang, China, and Kazakhstan to advance our understanding of the main distribution characteristics and patterns of different types of locusts for effective locust monitoring and management.

Various factors influence the species distribution model (GCM) projections, including the type of data and environmental variables and the type of species distribution model. The limitations of our study depend largely on the nature of the data themselves and the interactions between environmental variables that affect locust distribution. In terms of data, although we tried to use as many sample points as possible to ensure an adequate sample size, most of the locust sample data came from field surveys, making it more difficult to obtain samples from Kazakhstan. Therefore, in order to eliminate the problem of low sample density in Kazakhstan, we used a plug-in for the spatially rarefy occurrence data of SDM Toolbox 2.0, Sparse Species Occurrence [63,64]. In terms of model constraints, the ecological niches of the three locusts we studied were slightly different [3,4,14,50,65]. However, the uneven sample size composition of the different species types in the sample may affect the results of the predicted distribution. The different proportions of the three locust species we studied in the total sample may also lead to differences in the distribution of typical locust suitability zones in Kazakhstan and China. We used Pearson correlation analysis and variance inflation factors to censor variables and eliminate autocorrelation and multicollinearity between environmental variables.

Other factors affecting locust success include other biotic and abiotic factors that constrain locust occurrence and survival. In addition, improvements in grassland ecology, biological predators of the grassland locust, competition between species for food resources, habitat type, land-use patterns, land cover, and other drivers affecting the potential distribution of locusts will have a dampening effect on large outbreaks. Therefore, the actual habitat area may be smaller. 

Our research work is based on three kinds of migratory locusts in a niche overlap and the geographic distribution of preliminary forecast of the locust, the geographical distribution of typical quantitative habitat suitability change, understanding the China–Kazakhstan border areas, the concentrated distribution area of typical locusts and its future expansion area. The scope of the initial assessment of typical locusts confirms the emphases of the locust control between the two countries in the region, to provide a basis for future monitoring work. Large-scale locust monitoring measures are difficult to implement in practice; therefore, this study will provide a regional monitoring focus for locust control efforts. Our study complements this by assessing priority areas for prevention. Although the ecological niches of the three locusts we studied are slightly different, the conclusions we draw are only intended to highlight the hotspots where the three locusts congregate, to point out their potential migration risk and to provide a preliminary assessment of the risk of locust occurrence. We are more interested in the migration potential of locusts between the two areas, especially in the border areas.

## 5. Conclusions

Our results indicated that the critical environmental variables affecting this species were the soil surface sand content, slope, mean precipitation during the hottest season, and precipitation seasonality (variable speed coefficient). The main distribution of the three typical locust species was in the upper Irtysh River region, the Alatao Mountain region, the northern slopes of the Tianshan Mountains, the Sayram Lake region, the eastern part of the Alakol Lake region, the Tekes River region, the western part of Ulungur Lake, the Ili River, the upper Tarim River region, and the A-erh-chin Mountains region. In addition, most of Kazakhstan’s regions are unsuitable for survival. Only a few areas of low suitability occur in Lake Zaysan, around Lake Balkhash, and the regions near Lake Alakol and the Aral Sea. Based on different climate scenarios, the potential habitat of the three typical locusts is expected to shrink over time; it is expected to decrease by 3.9 × 10^4^–4.6 × 10^4^ km^2^ in the 2030s and by 6.4 × 10^4^–10.6 × 10^4^ km^2^ in the 2050s. However, the areas of high suitability for locusts showed a brief expansion, increasing by 1 × 10^4^–0.4 × 10^4^ km^2^ in the 2030s. Although there was an overall downwards trend in the area potentially suitable for locust survival and a significant decrease in moderate- and low-suitability areas for locusts, the high-suitability areas increased. The dynamic monitoring and risk assessment will still need to be reinforced in these areas.

## Figures and Tables

**Table 1:** VIF for each environmental variable.

Variable	Description	VIF
Bio1	Annual Mean Temperature (°C)	3.155
Bio2	Mean Diurnal Range (mean of monthly (max temp−min temp))	1.402
Bio4	Temperature Seasonality (standard deviation × 100)	4.956
Bio9	Mean Temperature of Driest Quarter (°C)	2.97
Bio14	Precipitation of Driest Month (mm)	8.408
Bio15	Precipitation Seasonality (coefficient of variation)	7.482
Bio18	Precipitation of Warmest Quarter (mm)	8.022
Slope	Slope (°)	1.074
T-SAND	Topsoil Sand Fraction (%wt.)	1.137

**Table 2:** Changes in the potential habitat area of typical locust species relative to the current area based on different climate scenarios.

Scenarios	Time	Low Suitable Area (×10^5^ km^2^)			Moderately Suitable Area (×10^5^ km^2^)			Highly Suitable Area (×10^5^ km^2^)		
		Gain	Loss	Unchanged	Gain	Loss	Unchanged	Gain	Loss	Unchanged
SSP126	2030s	0.94	1.20	1.63	0.32	0.52	0.67	0.15	0.13	0.33
	2050s	0.92	1.22	1.61	0.31	0.56	0.63	0.15	0.15	0.32
SSP245	2030s	0.96	1.30	1.53	0.36	0.53	0.66	0.17	0.13	0.34
	2050s	0.96	1.28	1.55	0.35	0.58	0.61	0.12	0.20	0.26
SSP370	2030s	0.91	1.23	1.60	0.32	0.51	0.68	0.15	0.13	0.34
	2050s	0.93	1.31	1.51	0.31	0.58	0.61	0.12	0.17	0.30
SSP585	2030s	0.97	1.17	1.66	0.35	0.52	0.67	0.15	0.14	0.32
	2050s	0.94	1.41	1.41	0.35	0.52	0.67	0.08	0.24	0.22

## Data Availability

All relevant data are included in the manuscript.

## Supplementary Materials

The following supporting information can be downloaded at: https://www.mdpi.com/article/10.3390/insects13100942/s1, Table S1: Initially selected environment variables; Table S2: Model performance under different regularization multiplier and feature combination.
