# Supplementary material for: Predictions Based on Different Climate Change Scenarios: The Habitat of Typical Locust Species Is Shrinking in Kazakhstan and Xinjiang, China
Source: Insects. 2022 Oct 17;13(10):942. doi: 10.3390/insects13100942 (PMC9603880; doi:10.3390/insects13100942)
Supplement: Supplementary file 1 [file insects-13-00942-s001.zip › insects-1917285-supplementary-done-done.pdf]

insects

Table S1. Initially selected environment variables.

Variable	Describe
Bio1	Annual mean temperature (℃)
Bio2	Monthly diurnal range (℃)
Bio3	Isothermally (%)
Bio4	Temperature seasonality (standard deviation ×100)
Bio5	Max temperature of the warmest month (℃)
Bio6	Min temperature of coldest month (°C)
Bio7	Annual range of temperature (℃)
Bio8	Mean temperature of wettest quarter (°c)
Bio9	Mean temperature of the driest quarter (℃)
Bio10	Mean temperature of warmest quarter (°c)
Bio11	Mean temperature of coldest quarter (°c)
Bio12	Annual Precipitation (mm)
Bio13	Precipitation of wettest month (mm)
Bio14	Precipitation of the driest month (mm)
Bio15	Precipitation seasonality (Coefficient of Variation)
Bio16	Precipitation of wettest quarter (mm)
Bio17	Precipitation of driest quarter (mm)
Bio18	Mean precipitation of the warmest quarter (mm)
Bio19	Mean precipitation of the coldest quarter (mm)
Alt	Altitude (m)
Slope	Slope (°)
Aspect	Aspect (°)
T-SAND	Topsoil Sand Fraction (% wt.)

Table S2. Model performance under different regularization multiplier and feature combination.

Regularization Multiplier	Feature Combination	Train AUC	Test AUC	TSS
0.5	H	0.935	0.927	0.7736
0.5	L	0.885	0.8799	0.6769
0.5	LQ	0.912	0.9141	0.7185
0.5	LQH	0.926	0.928	0.7587
0.5	LQHP	0.936	0.923	0.774
0.5	LQHPT	0.955	0.931	0.8115
1	H	0.933	0.9234	0.7662
1	L	0.874	0.8782	0.6476
1	LQ	0.914	0.9132	0.7211
1	LQH	0.948	0.9436	0.7906
1	LQHP	0.933	0.9183	0.7651
**1**	LQHPT	0.942	0.9303	0.786
1.5	H	0.930	0.9248	0.7539

1.5	L	0.886	0.8842	0.6734
1.5	LQ	0.910	0.9073	0.7098
1.5	LQH	0.929	0.9245	0.7573
1.5	LQHP	0.930	0.9236	0.7609
1.5	LQHPT	0.932	0.9265	0.7644
2	H	0.924	0.9202	0.6555
2	L	0.879	0.8794	0.6555
2	LQ	0.910	0.912	0.7157
2	LQH	0.927	0.9245	0.7495
2	LQHP	0.928	0.9262	0.752
2	LQHPT	0.932	0.9267	0.763
2.5	H	0.928	0.9181	0.7512
2.5	L	0.890	0.881	0.6963
2.5	LQ	0.913	0.9067	0.7207
2.5	LQH	0.925	0.9203	0.7426
2.5	LQHP	0.927	0.9241	0.709
2.5	LQHPT	0.949	0.9491	0.793
3	H	0.925	0.9227	0.7566
3	L	0.883	0.8771	0.6683
3	LQ	0.911	0.9054	0.7103
3	LQH	0.925	0.9229	0.7465
3	LQHP	0.943	0.9398	0.7548
3	LQHPT	0.929	0.9223	0.7477
3.5	H	0.922	0.9182	0.7366
3.5	L	0.879	0.8859	0.703
3.5	LQ	0.905	0.903	0.7082
3.5	LQH	0.922	0.9185	0.75
3.5	LQHP	0.922	0.9211	0.7444
3.5	LQHPT	0.922	0.9204	0.7514
4	H	0.923	0.9194	0.7385
4	L	0.876	0.873	0.6705
4	LQ	0.906	0.909	0.7223
4	LQH	0.925	0.9172	0.7458
4	LQHP	0.925	0.9228	0.7577
4	LQHPT	0.924	0.9235	0.7538
4.5	H	0.923	0.9181	0.7418
4.5	L	0.874	0.8695	0.6627
4.5	LQ	0.908	0.9081	0.7164
4.5	LQH	0.924	0.9219	0.757
4.5	LQHP	0.943	0.9401	0.783
4.5	LQHPT	0.928	0.9223	0.7435
5	H	0.922	0.9231	0.7571

5	L	0.875	0.874	0.6741
5	LQ	0.911	0.9035	0.7185
5	LQH	0.924	0.9209	0.7454
5	LQHP	0.921	0.9154	0.7374
5	LQHPT	0.925	0.9213	0.7442
5.5	H	0.919	0.9219	0.759
5.5	L	0.878	0.8813	0.6844
5.5	LQ	0.908	0.9018	0.7145
5.5	LQH	0.924	0.9212	0.7529
5.5	LQHP	0.922	0.9221	0.7283
5.5	LQHPT	0.923	0.9196	0.7461
6	H	0.921	0.924	0.7621
6	L	0.872	0.8737	0.6766
6	LQ	0.905	0.9011	0.7073
6	LQH	0.922	0.9011	0.7409
6	LQHP	0.942	0.9411	0.7885
6	LQHPT	0.924	0.921	0.7469
6.5	H	0.921	0.9186	0.7459
6.5	L	0.873	0.8696	0.6627
6.5	LQ	0.905	0.9122	0.7422
6.5	LQH	0.923	0.9228	0.7489
6.5	LQHP	0.921	0.9201	0.7504
6.5	LQHPT	0.924	0.9174	0.7423
7	H	0.921	0.9149	0.7447
7	L	0.873	0.8773	0.6844
7	LQ	0.903	0.9004	0.7049
7	LQH	0.921	0.9231	0.7455
7	LQHP	0.921	0.9199	0.7398
7	LQHPT	0.919	0.9093	0.7331
7.5	H	0.920	0.9189	0.7525
7.5	L	0.886	0.8871	0.6997
7.5	LQ	0.922	0.9205	0.7343
7.5	LQH	0.939	0.939	0.7725
7.5	LQHP	0.922	0.918	0.7455
7.5	LQHPT	0.924	0.9187	0.7477
8	H	0.921	0.9215	0.7475
8	L	0.870	0.8644	0.6522
8	LQ	0.907	0.9051	0.7231
8	LQH	0.92	0.9173	0.7421
8	LQHP	0.922	0.9197	0.7426
8	LQHPT	0.921	0.918	0.7404
8.5	H	0.92	0.9179	0.7502

8.5	L	0.867	0.8691	0.6763
8.5	LQ	0.923	0.9256	0.7467
8.5	LQH	0.918	0.9162	0.732
8.5	LQHP	0.938	0.9351	0.7694
8.5	LQHPT	0.924	0.9227	0.7512
9	H	0.916	0.9165	0.7489
9	L	0.872	0.8641	0.661
9	LQ	0.921	0.903	0.7384
9	LQH	0.919	0.9166	0.7436
9	LQHP	0.921	0.9194	0.7471
9	LQHPT	0.922	0.92	0.7588
9.5	H	0.862	0.919	0.6549
9.5	L	0.892	0.8609	0.6924
9.5	LQ	0.936	0.8965	0.7649
9.5	LQH	0.922	0.9318	0.7461
9.5	LQHP	0.921	0.9231	0.7376
9.5	LQHPT	0.920	0.9173	0.7477
10	H	0.870	0.9184	0.6758
10	L	0.899	0.866	0.6956
10	LQ	0.920	0.8959	0.7417
10	LQH	0.919	0.9182	0.7342
10	LQHP	0.9186	0.9148	0.7511
10	LQHPT	0.921	0.9175	0.7384

Note: L = linear, Q = quadratic, H = hinge, P = product, and T = threshold.
